# The efficacy of intraoperative periarticular injection in Total hip arthroplasty: a systematic review and meta-analysis

**DOI:** 10.1186/s12891-019-2628-7

**Published:** 2019-06-01

**Authors:** Hsuan-Hsiao Ma, Te-Feng Arthur Chou, Shang-Wen Tsai, Cheng-Fong Chen, Po-Kuei Wu, Wei-Ming Chen

**Affiliations:** 1 Department of Orthopaedics and Traumatology, Taipei Veterans General Hospital, Taipei, Taiwan; 2 Department of Surgery, School of Medicine, National Yang-Ming University, Taipei, Taiwan

**Keywords:** Local infiltration analgesics, Pain management, Periarticular injection, Total hip arthroplasty

## Abstract

**Background:**

Periarticular injection (PAI) is a regional analgesia method performed in total hip arthroplasty (THA) for postoperative pain relief. However, its efficacy and safety is still inconclusive. Therefore, we conducted this meta-analysis to assess the safety of PAI and to determine if PAI provides better pain relief and reduce the consumption of opioids after THA.

**Methods:**

We searched PubMed, Web of Science, Embase and the Cochrane Library comprehensively. Only randomized control trials were included in our meta-analysis. Eleven studies that compared the efficacy of PAI with the control group were included. The preferred reporting items for systematic reviews and meta-analysis (PRISMA) guidelines and Jadad score were applied to assess the quality of the included studies. We used the recommendations by the Cochrane Collaboration to reduce bias and to ensure our results were reliable and veritable.

**Results:**

Our analysis demonstrated that PAI was more effective than the control group with a lower visual analog scale (VAS) score during rest at 24 h (*P* = 0.003), 48 h (*P* = 0.002), and VAS score with activity at 24 h (*P* = 0.04). There was also less amount of opioid consumption (*P* = 0.01). There were no differences in length of hospital stay (*P* = 0.526) and postoperative nausea rate (*P* = 0.153).

**Conclusion:**

Compared with the control group, PAI showed better pain relief and less amount of opioid consumption after THA. Our meta-analysis suggests that PAI is a safe and effective multimodal analgesia technique that can be used for THA.

**Electronic supplementary material:**

The online version of this article (10.1186/s12891-019-2628-7) contains supplementary material, which is available to authorized users.

## Background

Postoperative pain management in patients who had undergone total hip arthroplasty (THA) continues to be an evolving issue for physicians. The primary goal is to reduce pain in the early postoperative period as well as reducing the amount of opioid consumption. Improved pain management can lead to accelerated mobilization and rehabilitation, fewer postoperative complications and shorter length of stay, which are the essential elements of a fast-track recovery program [1–3]. In addition, adverse effects associated with opioid consumption, including nausea, vomiting, respiratory depression, hypotension, decreased gastrointestinal mobility and urinary retention can be reduced if less opioid were administered [4].

Periarticular injection (PAI) (also known as local infiltration analgesia or periarticular multimodal drug injection) is a new, alternative regional analgesic that involves administering analgesics into the surrounding tissue in the surgical field. This method usually consists of a local anesthetics of amide derivatives (eg, ropivacaine, bupivacaine, levobupivacaine, ropivacaine) and/or corticosteroids, opioids, epinephrine, nonsteroidal anti-inflammatory drugs and dilution with normal saline. In terms of its efficacy, several studies have validated the benefit of PAI on post-operative pain relief [5–9], while some other studies did not find an improvement in pain control [10–15]. Therefore, the efficacy of PAI in THA remains inconclusive. The present meta-analysis was conducted to determine the efficacy of PAI as a postoperative pain management in patients who had undergone THA surgery.

## Methods

### Search strategy

**Fig. 1:**
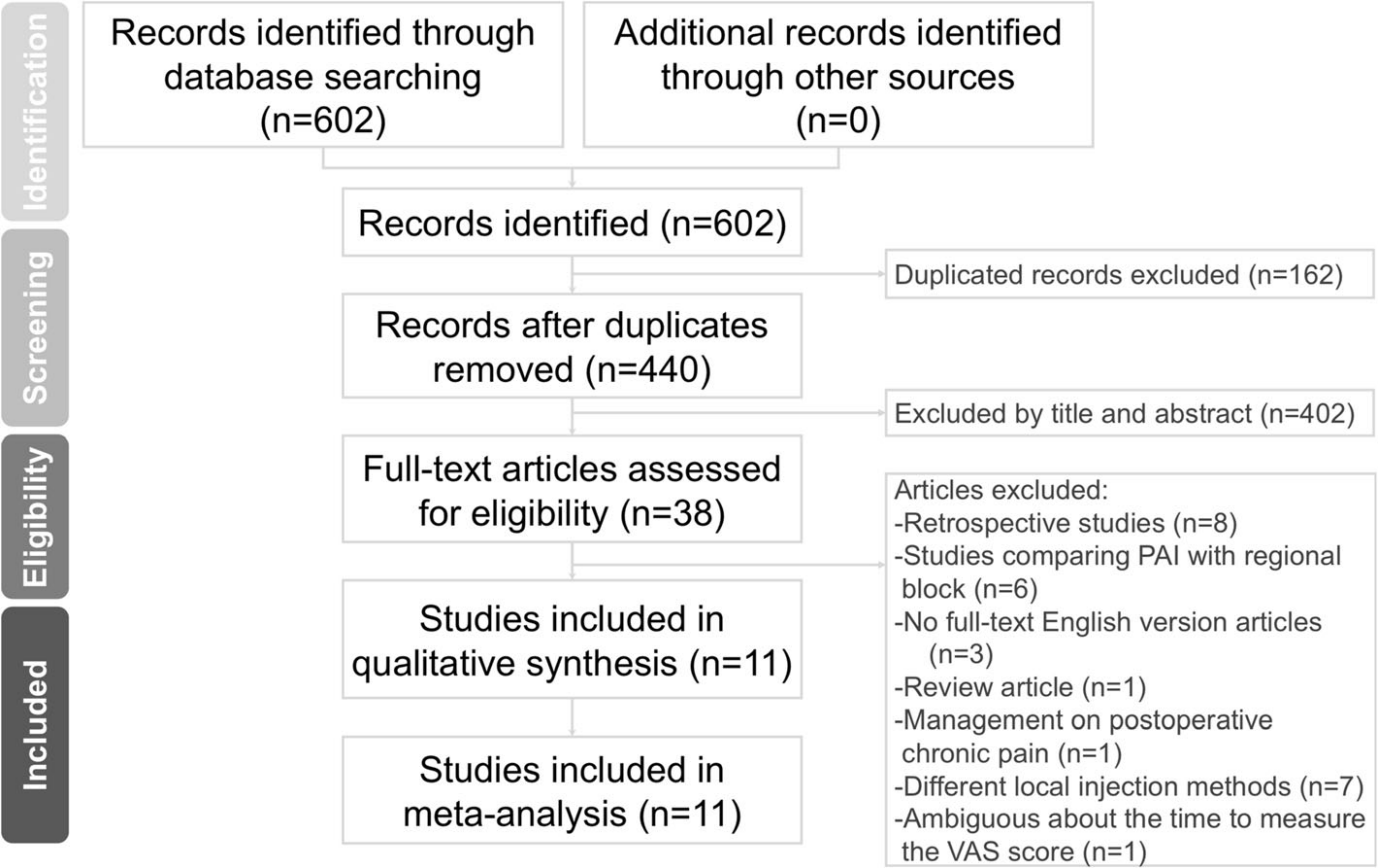
Preferred reporting items for systematic reviews and meta-analysis (PRISMA) flow diagram for the searching and identification of included studies

We identified randomized controlled trials (RCTs) conducted for PAI in THA by searching databases including PubMed, Web of Science, Embase and the Cochrane Library from the earliest record to October 2018. The bibliographies of the included studies were manually reviewed for relevant references. Studies not written in English or not available in full text were excluded. We investigated studies employing PAI for the relief of hip pain in patients who had undergone THA. The search strategy comprised the following keywords in variable combination: (total hip arthroplasty OR total hip replacement) AND (local infiltration OR periarticular injection OR periarticular infiltration). Regarding the types of included studies, we enrolled only randomized controlled trials (RCTs) and excluded comparative experimental trials, single-armed follow-up studies, case series and case reports. All identified studies were required to comprise at least two treatment arms, one of which was PAI and the other was placebo injection or no injection. The search strategy is presented in Fig. 1.

### Inclusion criteria

We considered studies that were eligible for meta-analysis if they met the PICOS criteria (population, intervention, comparator, outcomes, study design). Population: patients who had undergone THA. Intervention: Using periarticular injection as the pain control method for THA. Comparator: Periarticular injection or those not using PAI or placebo (control group). Outcomes: Visual analog scale (VAS) at different time points, amount of opioid consumption, length of hospital stay. Studies must have a follow-up rate of at least 90%, and at least 1 of the above outcome parameters must be included. Study design: interventional randomized controlled trials.

### Data extraction and quality assessment

Two reviewers examined all the identified articles and extracted data using a predetermined form. We recorded the first author, year, study design, enrolled sample number, type of treatment arms, type of anesthesia, regimen of drug infiltration, outcome parameters to assess pain, function, length of hospital stay and nausea after THA. Two reviewers independently evaluated the methodological quality of the enrolled studies using Jadad score for the RCTs (Table 1). There are three aspects in Jadad score to evaluate the methodology of RCTs including: randomization, blinding and an account of all patients. The score ranged from 0 to 5; a higher score indicated better methodological quality. Discrepancies between the two reviewers were solved after thorough discussion.

**Table 1:** Characteristics of included studies

Author, year	Study design	Enrolled Sample number (G1/G2)	Comparing	Anesthesia	Drug infiltration	Outcome measurement						Quality assessment^a^
						a	b	c	d	e	f	
Hirasawa, 2018 [14]	RCT	45/45	PAI, no PAI	GA	Rop 300 mg, Mor 8 mg, Methyl 40 mg, Keto 50 mg, Epi 0.3 mg	V		V				5
Ban WR,2017 [5]	RCT	43/43	PAI, no PAI	GA	Rop200mg, Keto 30 mg, Epi 0.3 mg	V		V		V		5
Villatte, 2016 [6]	RCT	75/75	PAI, no PAI	GA	Rop 235 mg, Epi 0.5 mg	V		V		V		5
Hofstad, 2015 [10]	RCT	55/54	PAI, no PAI	SA	Rop 300 mg, Epi 0.5 mg				V	V		5
den Hartog, 2015 [11]	RCT	25/25	PAI, no PAI	SA	Rop 200 mg, Epi 0.5 mg					V	V	5
Zoric, 2014 [12]	RCT	29/29	PAI, no PAI	GA	Rop 160 mg			V	V		V	4
Dobie, 2012 [15]	RCT	46/46	PAI, no PAI	GA	Levo 160 mg	V				V		5
Murphy, 2012 [7]	RCT	45/46	PAI, no PAI	SA	Bup 150 mg	V		V	V			5
Lunn, 2011 [13]	RCT	60/60	PAI, no PAI	SA	Rop 200 mg, Epi 1.5 mg					V		5
Liu, 2011 [8]	RCT	40/40	PAI, no PAI	SA	Bup 30 mg, Mor 5 mg, Bet 1 mg, Epi 0.5 mg	V					V	5
Busch, 2010 [9]	RCT	32/31	PAI, no PAI	SA or GA	Rop 40 mg, Keto 30 mg, Epi 0.5 mg	V						4

G1 group: study group (PAI); G2 Group: control group (no PAI)

Outcome measure: a = VAS at rest at 24 h, b = VAS at activity at 24 h, c = VAS at rest at 48 h, d = total additional morphine consumption in post-op 24 h, e = Length of hospital stay, f = nausea

*Bup* bupivacaine, *Epi* epinephrine, *Keto* ketorolac, *Mor* morphine, *Rop* ropivacaine, *Levo* levobupivacaine, *Bet* betamethasone, *Methyl* methylprednisolone

^a^Jadad score

### Evaluation of publication bias

A thorough risk-of-bias assessment was completed to identify factors that may have altered the results of this analysis. Two senior reviewers independently evaluated each included study and documented their potential for selection bias, performance bias, detection bias, attrition bias, and reporting bias using the Cochrane tool for assessing risk of bias in randomized trials. Funnel plots were constructed to visually detect the presence of publication bias.

### Data synthesis

The standardized mean differences (SMDs) of postoperative VAS score at different time points between the PAI and control group was the primary outcome. Data were extracted from the VAS score at 24 h and 48 h, amount of opioid consumption. A negative SMD value indicated PAI to be a favorable treatment option. The SMDs of length of hospital stay, amount of opioid consumption and odds ratios (ORs) of post-operative nausea in the PAI group compared with the control group comprised of the secondary outcome. A random effect model was utilized to pool individual SMDs and ORs. Analyses were performed using Comprehensive Meta-Analysis (CMA) software, version 3 (Biostat, Englewood, NJ, USA). Between-trial heterogeneity was determined by using *I*2 tests; values > 50% were regarded as considerable heterogeneity. Funnel plots and Egger’s test were used to examine potential publication bias. Statistical significance was defined as *p*-values < 0.05.

## Results

### Search results

We identified 602 relevant articles according to the search strategy. One-hundred and sixty-two duplicate records were removed using Endnote software. Four-hundred and two studies were excluded after reading the title and abstract. According to the inclusion criteria, 27 studies were excluded after reading the full article. Finally, 11 articles that compared the efficacy of periarticular injection in THA with those without were included for our meta-analysis. The baseline characteristics of the 11 included studies are summarized in Table 1. All of them were randomized controlled trials. The Jadad score of the 11 RCTs indicated that the studies were of high quality (4–5 points).

### Meta-analysis results

#### VAS score at 24 and 48 h

**Fig. 2:**
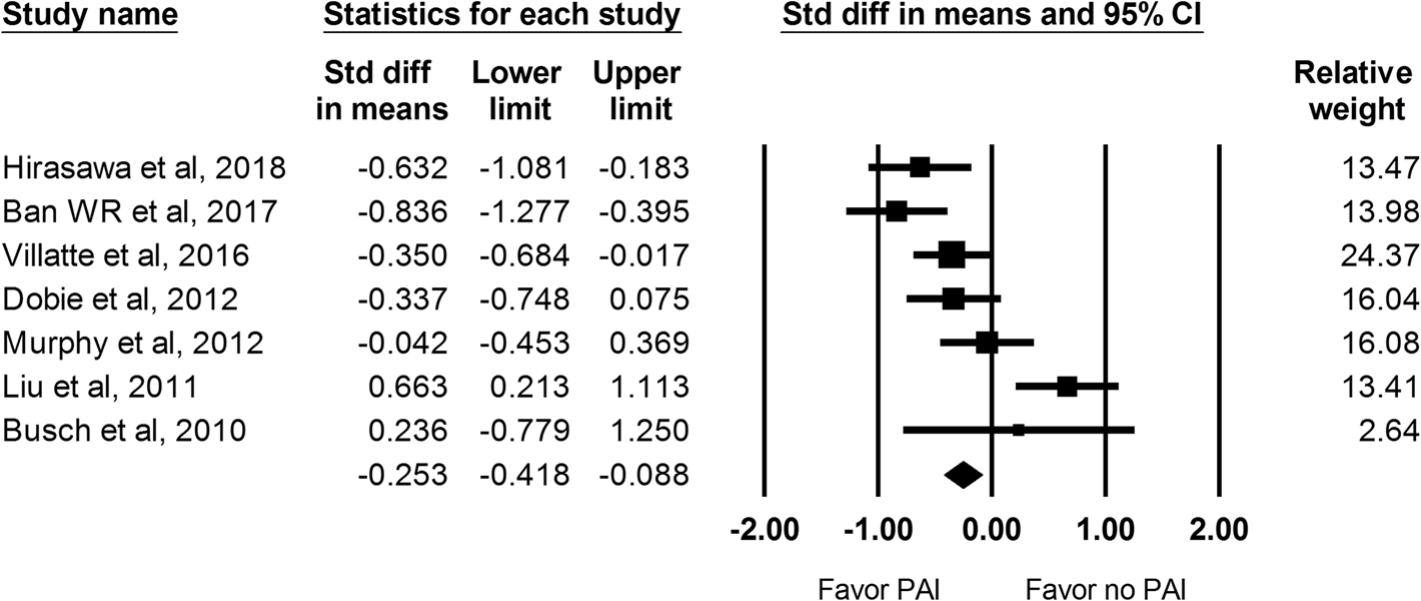
The effect of periarticular injection (PAI) on VAS score during rest at 24 h as compared with control group

The VAS score during rest at 24 h was recorded in 7 studies including a total of 652 hips. The analysis showed a significant lower VAS score at 24 h in the PAI group in comparison with the control group (SMD: -0.253; 95% CI − 0.418 to − 0.088; Fig. 2).

**Fig. 3:**
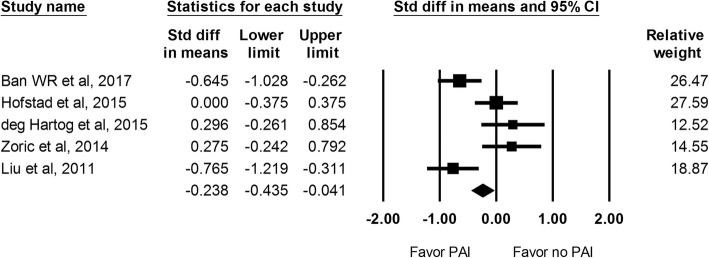
The effect of periarticular injection (PAI) on VAS score with activity at 24 h as compared with control group

VAS score with activity at 24 h was reported in 5 studies including 383 hips. There was an improved VAS score with activity noted in the PAI group than the control group (SMD -0.238; 95% CI − 0.435 to − 0.041; Fig. 3).

**Fig. 4:**
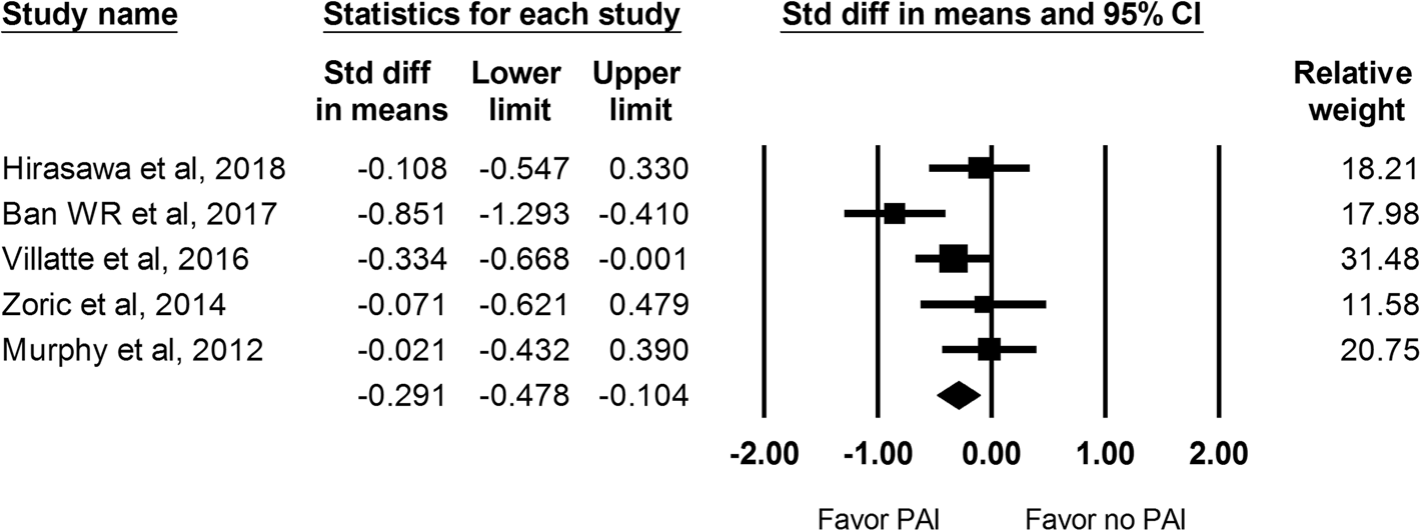
The effect of periarticular injection (PAI) on VAS score during rest at 48 h as compared with control group

There were 5 studies including 475 hips that reported the VAS score during rest at 48 h. There was also a lower VAS score during rest at 48 h noted in the PAI group versus the control group (SMD: -0.291; 95% CI − 0.478 to − 0.104; Fig. 4).

#### Amount of opioid consumption at 24 h

**Fig. 5:**
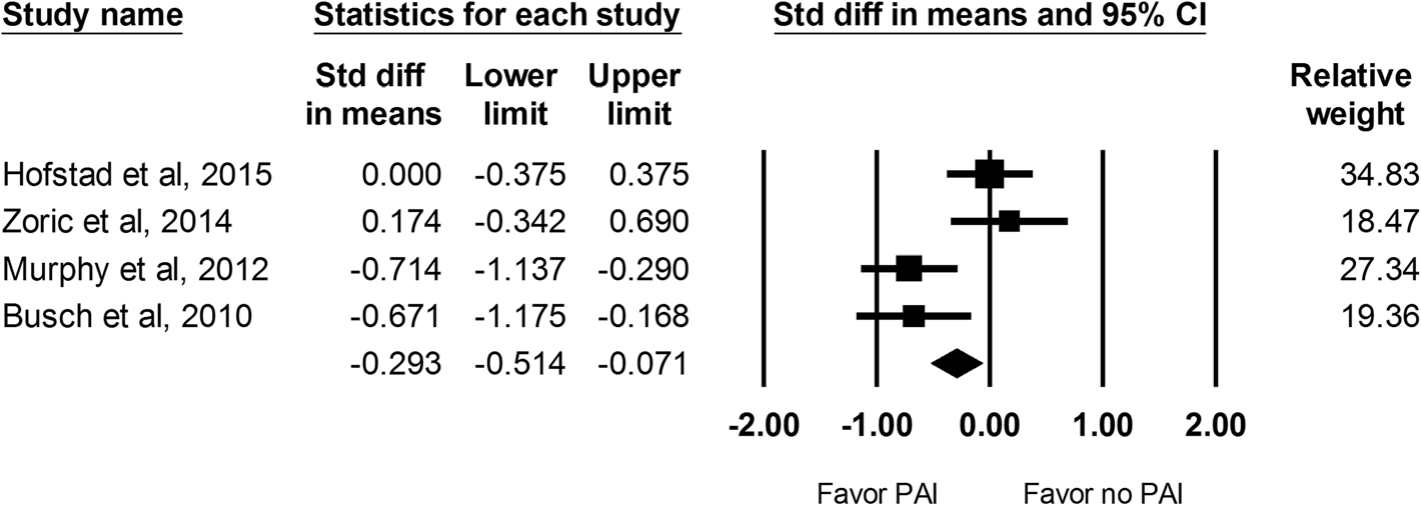
The effect of periarticular injection (PAI) on amount of opioid consumption at 24 h as compared with control group

Amount of opioid consumption during the hospital stay period was reported in 4 studies which included 321 hips. The results indicated patients that received PAI was associated with less amount of opioid consumption (SMD: -0.293; 95% CI − 0.514 to − 0.071; Fig. 5).

#### Length of hospital stay

**Fig. 6:**
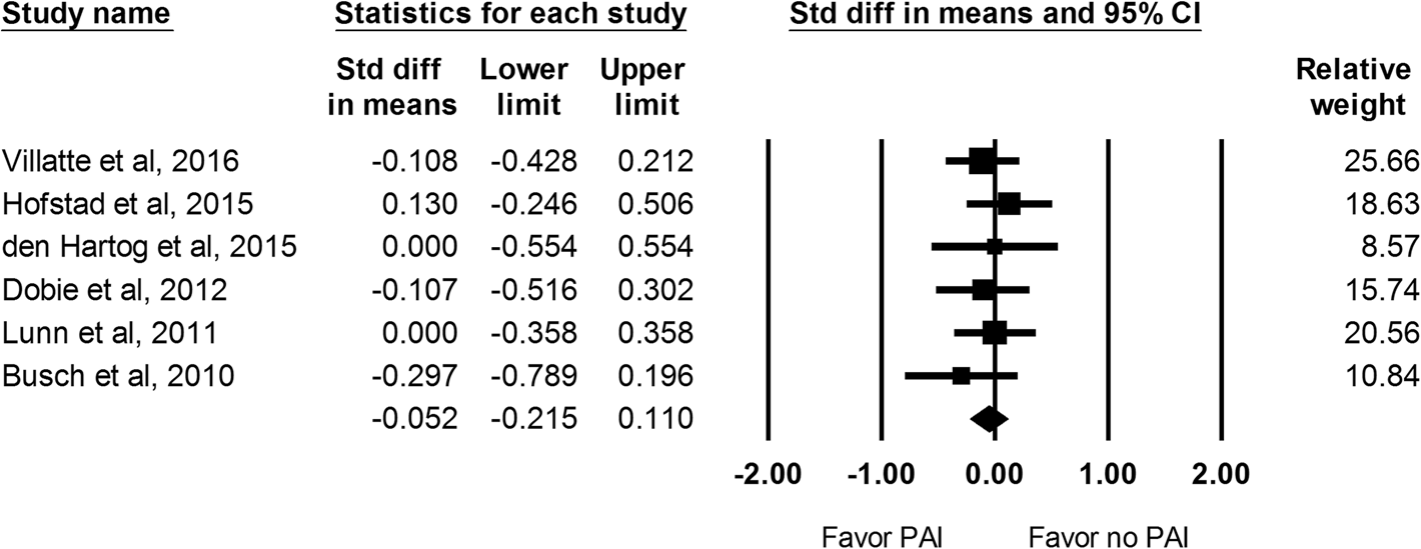
The effect of periarticular injection (PAI) on length of hospital stay at 24 h as compared with control group

There were 6 studies that recorded the length of hospital stay and a total of 584 hips. No significant difference was observed between the 2 groups (SMD: -0.052; 95% CI − 0.215 to 0.110; Fig. 6).

#### Postoperative nausea

**Fig. 7:**
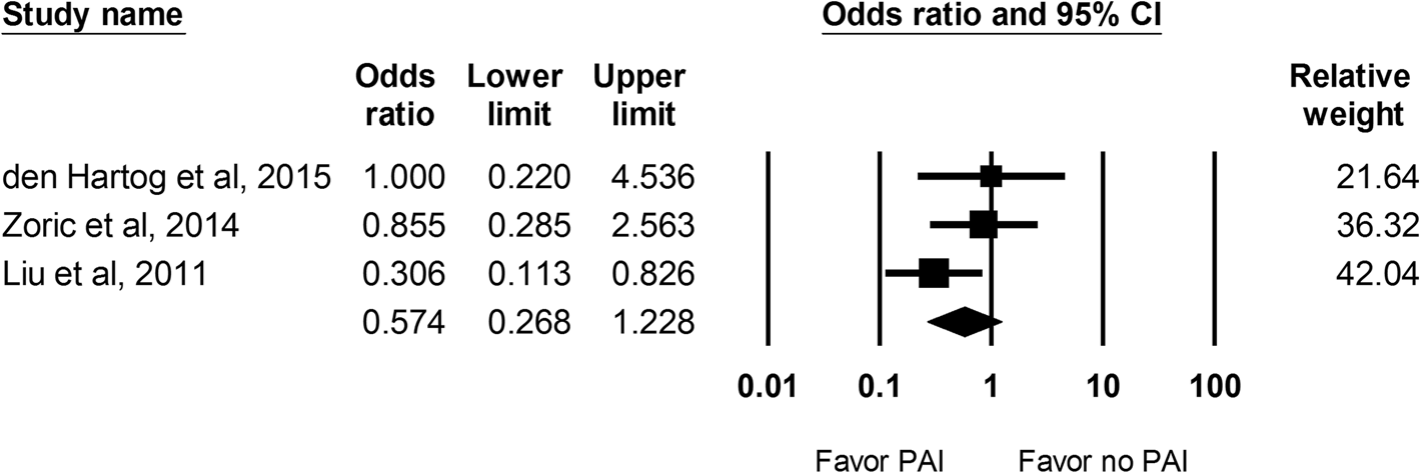
The effect of periarticular injection (PAI) on post-operative nausea at 24 h as compared with control group

The incidence of postoperative nausea was reported in 3 studies and a total of 188 hips were evaluated. No significant difference was found between the PAI and the control group (OR: 0.574; 95% CI 0.268 to 1.228; Fig. 7).

#### Adverse events and complications

The adverse events and complications of the enrolled studies were extracted and all the enrolled studies revealed no significant difference in the number of adverse events and complications (Table 2).

**Table 2:** Descriptions of complications in the study group of the included studies

Author, year	Enrolled Sample number (G1/G2)	Descriptions of complications in study group	Conclusion
Hirasawa, 2018 [14]	45/45	No complication including surgical site infection, wound complications, prolonged numbness or weakness, or allergy to the medication	No difference
Ban WR,2017 [5]	43/43	No mention	N/A
Villatte, 2016 [6]	75/75	Wound septic complication (2 hips, treatment group)	No difference
Hofstad, 2015 [10]	55/54	No mention	N/A
den Hartog, 2015 [11]	25/25	Dizziness, hypotension and retention of urine	No difference
Zoric, 2014 [12]	29/29	Nausea and vomiting	No difference
Dobie, 2012 [15]	46/46	Hypotension, chest pain, dural leak	No difference
Murphy, 2012 [7]	45/46	Urinary retention	No difference
Andersen, 2011 [16]	12/12	No clinical side effect, including cardiac and hemodynamic changes	No difference
Lunn, 2011 [13]	60/60	Quadriceps muscle palsy	No difference
Liu, 2011 [8]	40/40	Hemodynamic change, nausea and vomiting, urinary retention, respiratory depression, rash, and deep vein thrombosis	No difference
Busch, 2010 [9]	32/31	Wound blister, prominent suture trimming and deep vein thrombosis (4 hips, 3 in the treatment group and 1 in the control group)	No difference

G1: study group(PAI); G2: control group (no PAI)

*N/A* non applicable

#### Publication bias

**Fig. 8:**
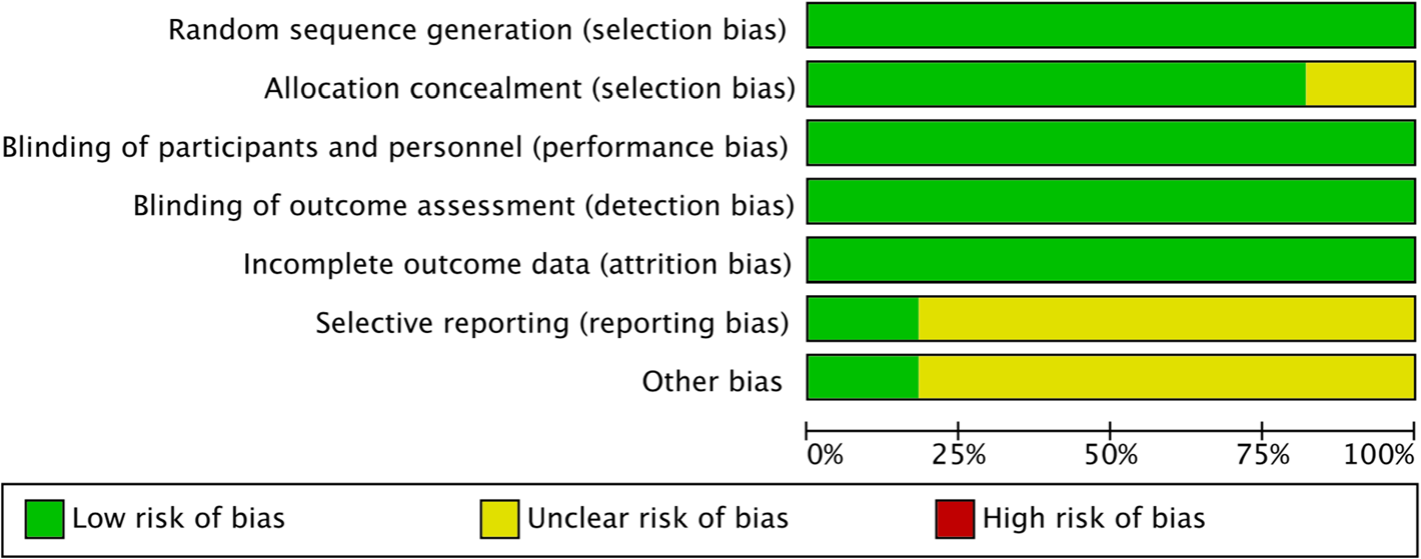
Summary of the assessment of the risk of bias

**Fig. 9:**
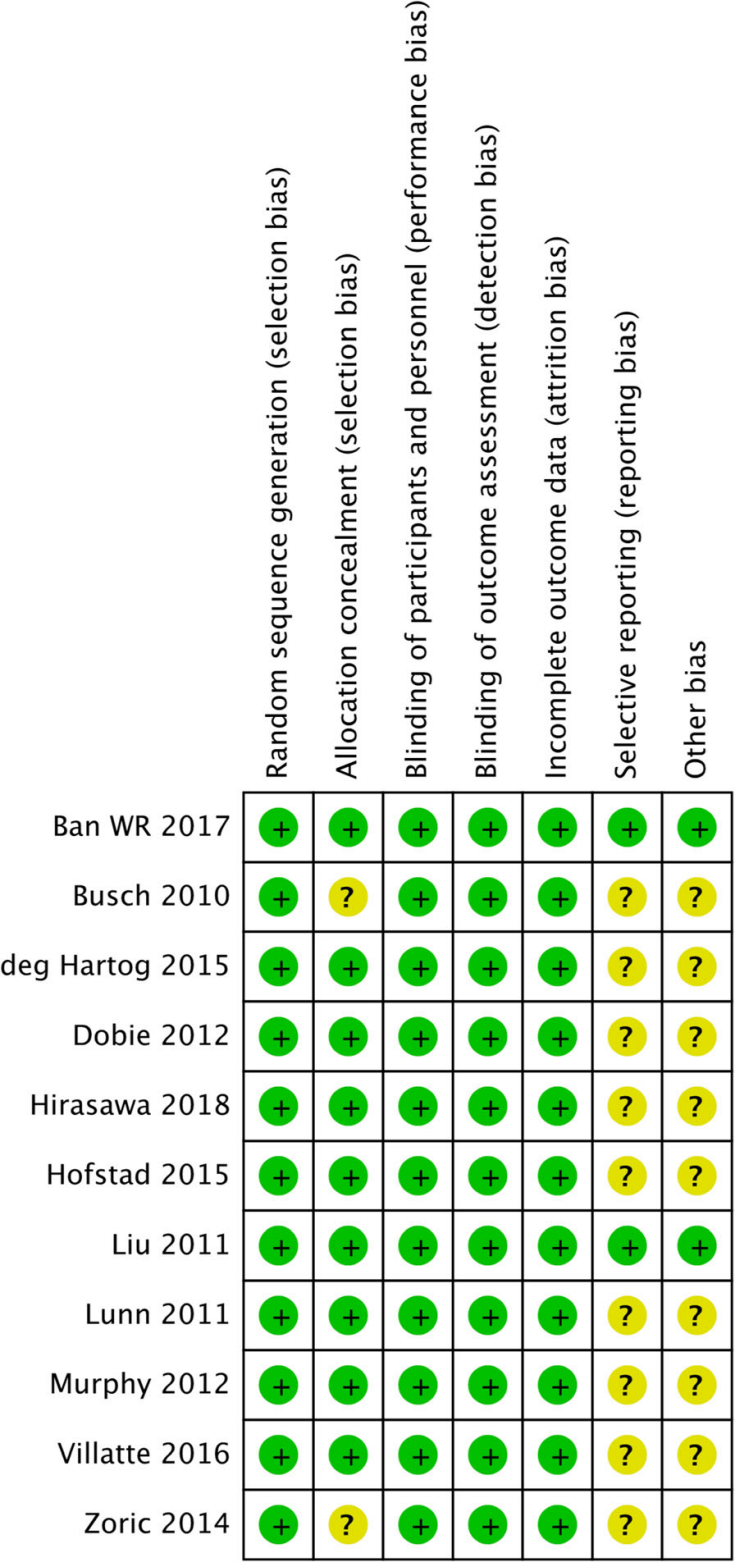
Results of risk of bias evaluation for each study according to the recommendations of the Cochrane Collaboration

Figures 8 and 9 summarizes the results of the risk of bias evaluation for each study. The allocation concealment bias (selection bias) was regarded as low except for 2 of the11 studies (18.2%). The completeness of the reported data (reporting bias) was unclear in 9 of the 11 (81.8%) studies included in this analysis. The Egger’s test revealed no significant publication bias regarding the overall SMD and odds ratio for the incidence of postoperative nausea. The funnel plots for SMD of all of the outcomes from each study and log odds ratio of postoperative nausea are shown. (Additional file 1: Figure S1, Additional file 2: Figure S2, Additional file 3: Figure S3, Additional file 4: Figure S4, Additional file 5: Figure S5 and Additional file 6: Figure S6).

## Discussion

The results of this meta-analysis focused on the efficacy of PAI in THA. It included 11 studies with 989 THA surgeries. In comparison with the control group, patients who received PAI presented with less pain (VAS score during rest at 24 and 48 h, VAS during activity at 24 h), and less amount of opioid consumption. Length of hospital stay and the incidence of postoperative nausea were not different.

Kehlet et al. [17] first introduced the term “multimodal” pain management, which involves the use of various medications that act on different regions of the pain pathway to achieve improved pain control [18, 19]. These medications can be administered either systemically or regionally. In recent literature, several regional analgesics have been utilized in total joint arthroplasty. For instance, the use of spinal anesthesia with intrathecal opioids, epidural anesthesia, nerve block and periarticular injection (PAI) are all commonly used techniques [18]. Parvizi et al. [18] has validated the use of spinal anesthesia with intrathecal opioids, epidural anesthesia and nerve block to be effective in pain relief but with adverse effects specific to each intervention [14]. Spinal anesthesia with intrathecal opioids was associated with increased risk of pruritus and gastrointestinal disturbance [20]. Epidural anesthesia might lead to pruritus, urinary retention and hypotension in the early post-operative period [21]. The use of femoral nerve block can potentially cause nerve damage and several reports have noted secondary infection in the setting of an indwelling catheter in patients who had undergone peripheral nerve block [22–24]. These adverse effects might lead to potential morbidities which makes PAI more appealing due to its limited adverse effects.

PAI has been widely studied for its potential efficacy in THA surgery. The potential benefits include pain relief during the acute postoperative period, improved functional recovery, reduced amount of opioid consumption, shortened length of hospital stay, lower rate of nausea and vomiting [5–16]. Jiang et al. [25] conducted a meta-analysis of using periarticular multimodal drug injection (PMDI) in TKA or THA patients and concluded the PMDI group was associated with improved pain relief, less opioid consumption, better range of motion and lower rate of nausea and vomiting. In the THA subgroup with a total of 5 studies included, PMDI group had lower VAS during rest at 6 h but was not associated with better outcomes in terms of VAS during rest at 24 h, opioid consumption at 24 h, length of hospital stay and wound complication rate. We excluded two studies from the analysis because additional PAI were given during the postoperative period. In one study, Specht et al. [26] conducted a randomized controlled study to validate the efficacy of multimodal drug infiltration administered through a catheter at 10 h and 22 h after surgery. In the study completed by Anderson et al. [27], the authors inserted an epidural catheter into the hip joint. Additional drug mixture was administered through the catheter on the morning of post-operative day 1.

Jiang et al. [25] included THA studies from up to November of 2011. After this meta-analysis, there were additional literature about the efficacy of PAI in THA and the results remain inconclusive [7, 10–13, 15, 16]. Therefore, Wang et al. [28] conducted a meta-analysis with a total of 666 THA patients from 8 randomized controlled trials to validate the efficacy of periarticular drug infiltration (PDI) for pain relief after THA surgery. The results showed that PDI group was associated with improved pain relief during rest but not with activity, less analgesic consumption and shorter length of hospital stay [24]. One of the 8 included study, Andersen et al. [16] inserted an epidural catheter and administered additional doses of drug mixture on 8 and 24 h after surgery, which was excluded from our analysis. In addition to the 8 studies included in this meta-analysis, Liu et al. [8] and Dobie et al. [15] conducted RCTs for PAI in THR in year 2011 and 2012, which should also be included for analysis. Wang et al. [28] searched results up to March 2016. According to our search results up to October 2018, there were additional literature with mixed results [1, 12]. Therefore, we conducted this meta-analysis for an update of efficacy of PAI in THA in terms of pain relief, total amount of opioid consumption, length of hospital stay and postoperative nausea.

The primary goal of PAI in THA is to relieve postoperative pain with direct, localized delivery of the drugs. Opioid rescue medication for the breakthrough pain was associated with adverse effects including nausea, vomiting, respiratory depression, hypotension, decreased gastrointestinal mobility and urinary retention [4]. Therefore, the secondary goal of PAI in THA is to reduce the total amount of opioid medication used in order to reduce systemic adverse effects. A mixture of local anesthetic and analgesics was meticulously infiltrated into the surrounding anatomic structures according to different surgical approaches. In general, an infiltration around the acetabulum, joint capsule, gluteus medius, gluteus minimus, tensor fascia lata and subcutaneous tissue was performed in a direct lateral approach [9, 11]. When using the posterolateral approach, drugs were administered around the acetabulum, joint capsule, short rotators, gluteus maximus, tensor fascia lata and subcutaneous tissue [7, 8, 12, 13, 15]. Injection of a mixture of medications into the surgical field might raise the concern of wound complications. Two studies reported wound complications in the PAI group. Busch et al. [9] had reported 3 patients with minor wound problems in the PAI group. The authors suggested that the wound problems might be related to their dressings or prominent sutures. Villatte et al. [6] had reported two septic complications in the PAI group that required subsequent debridement and irrigation. However, there were no apparent evidence suggesting that the technique used for these patients were direct causes of the infection. Moreover, the overall rate of wound complication was not different between the PAI group and control group.

This study is currently the most comprehensive meta-analysis to assess the efficacy of PAI in THA. However, there are several limitations that should be recognized. First, we searched only for English articles but not articles in other languages or unpublished data. This might be the potential source of publication bias. Second, heterogeneity of clinical settings between studies should be recognized, including regimens and doses of PAI, types of anesthesia and surgical approaches. Finally, several outcome parameters with clinical importance such as incidence of wound complications or acute periprosthetic joint infection, functional scores and range of motion of hip joint could not be evaluated in our meta-analysis because the incidence of such outcome parameter were rarely reported. Therefore, future studies can place an emphasis in determining the incidence of such adverse events to provide a more comprehensive result.

## Conclusions

In summary, the present meta-analysis revealed that PAI can lead to better pain relief and less amount of opioid consumption for patients following THA surgery. However, length of hospital stay and incidence of postoperative nausea were not different. Therefore, our meta-analysis suggests that PAI is a safe option for pain management in THA surgery. Future studies can focus on the functional score and the incidence of adverse events such as acute or late periprosthetic joint infection to provide a more comprehensive result.

## Additional files

Additional file 1: **Figure S1.** Funnel plot of VAS score during rest at 24 h. (TIF 5878 kb)

Additional file 2: **Figure S2.** Funnel plot of VAS score with activity at 24 h. (TIF 5872 kb)

Additional file 3: **Figure S3.** Funnel plot of VAS score during rest at 48 h. (TIF 5966 kb)

Additional file 4: **Figure S4.** Funnel plot of amount of opioid consumption at 24 h. (TIF 6106 kb)

Additional file 5: **Figure S5.** Funnel plot of length of hospital stay. (TIF 6146 kb)

Additional file 6: **Figure S6.** Funnel plot of post-operative nausea. (TIF 5837 kb)

## Abbreviations

OR: Odds ratio
PAI: Periarticular injection
PMDI: Periarticular multimodal drug injection
PRISMA: Preferred reporting items for systematic reviews and meta-analysis
RCT: Randomized controlled trial
SMD: Standardized mean difference
THA: Total hip arthroplasty
VAS: Visual analog scale
