# Supplementary figures and images for: The efficacy of intraoperative periarticular injection in Total hip arthroplasty: a systematic review and meta-analysis
Source: BMC Musculoskelet Disord. 2019 Jun 1;20:269. doi: 10.1186/s12891-019-2628-7 (PMC6545218; doi:10.1186/s12891-019-2628-7)

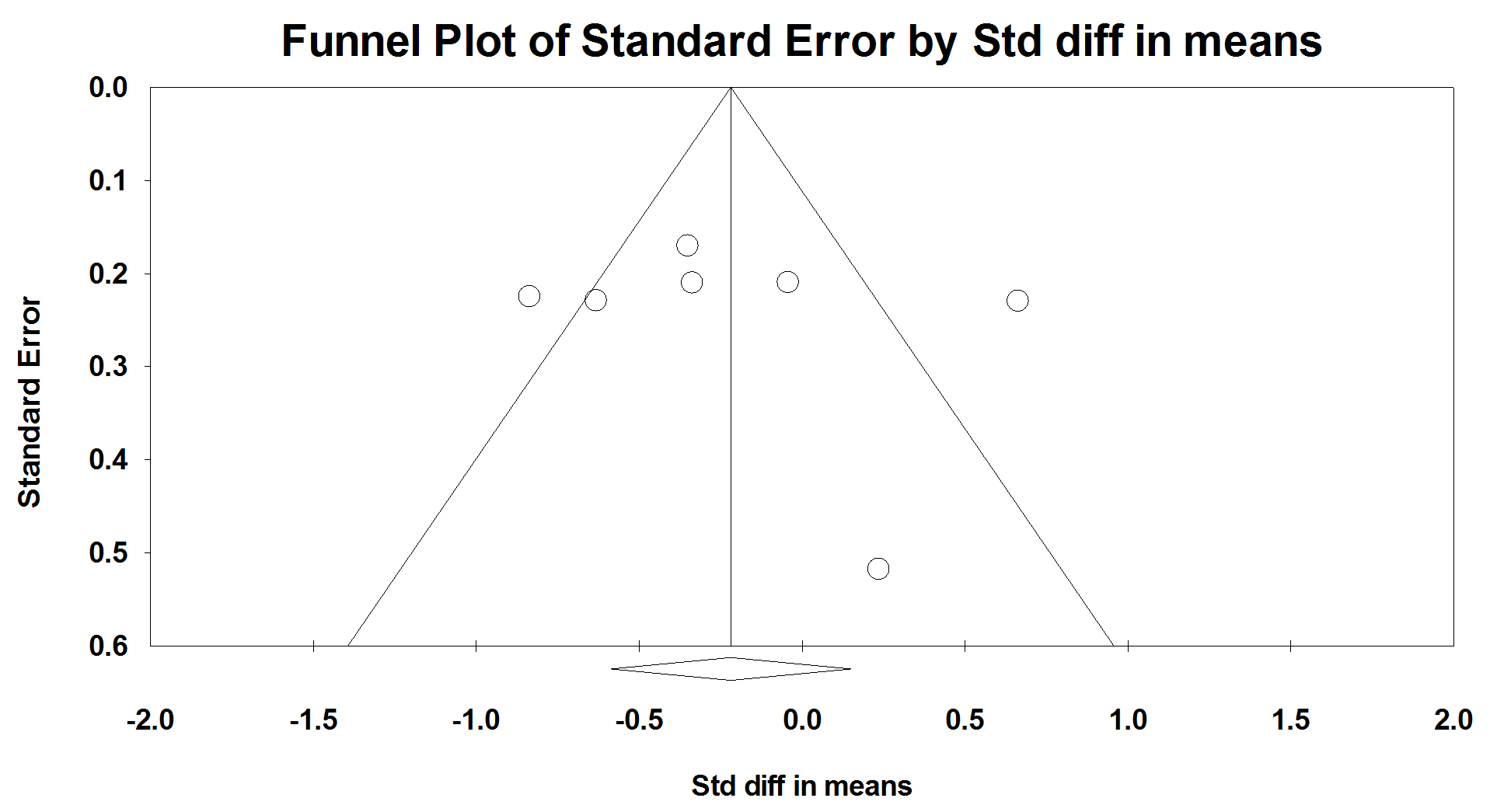

Supplement: Supplementary file 1 — Figure S1. Funnel plot of VAS score during rest at 24 h. (TIF 5878 kb) [file 12891_2019_2628_MOESM1_ESM.tif]

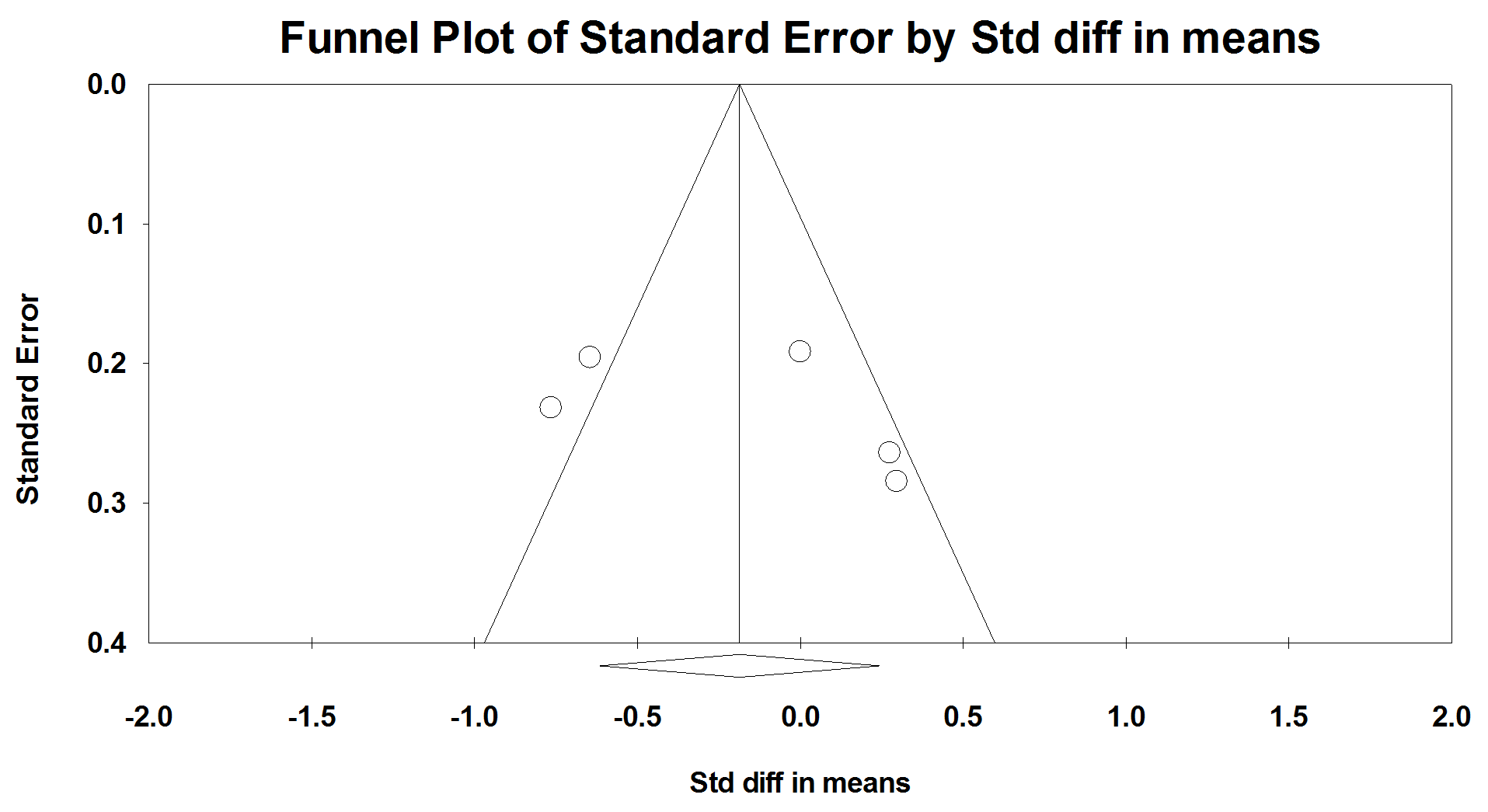

Supplement: Supplementary file 2 — Figure S2. Funnel plot of VAS score with activity at 24 h. (TIF 5872 kb) [file 12891_2019_2628_MOESM2_ESM.tif]

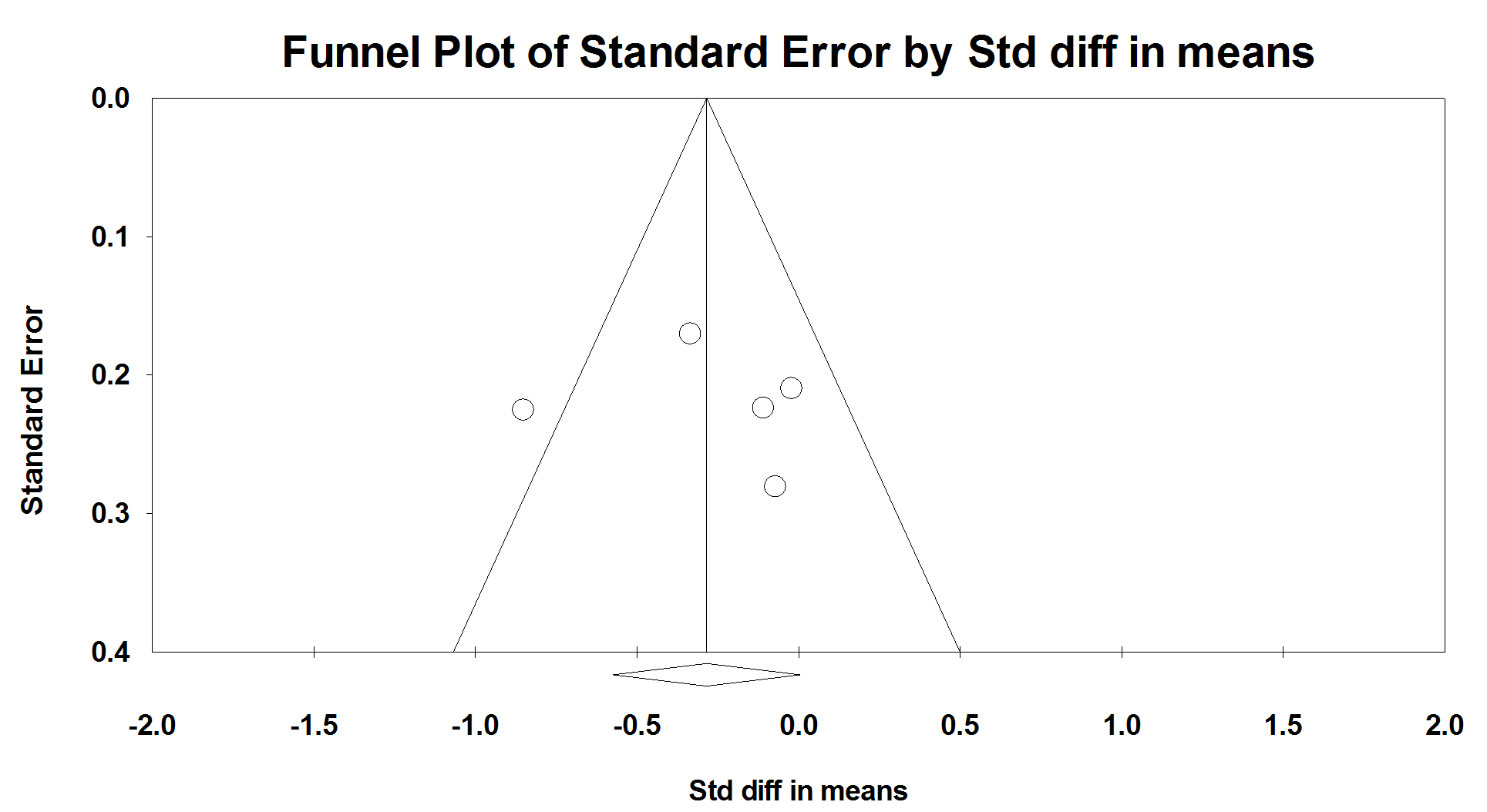

Supplement: Supplementary file 3 — Figure S3. Funnel plot of VAS score during rest at 48 h. (TIF 5966 kb) [file 12891_2019_2628_MOESM3_ESM.tif]

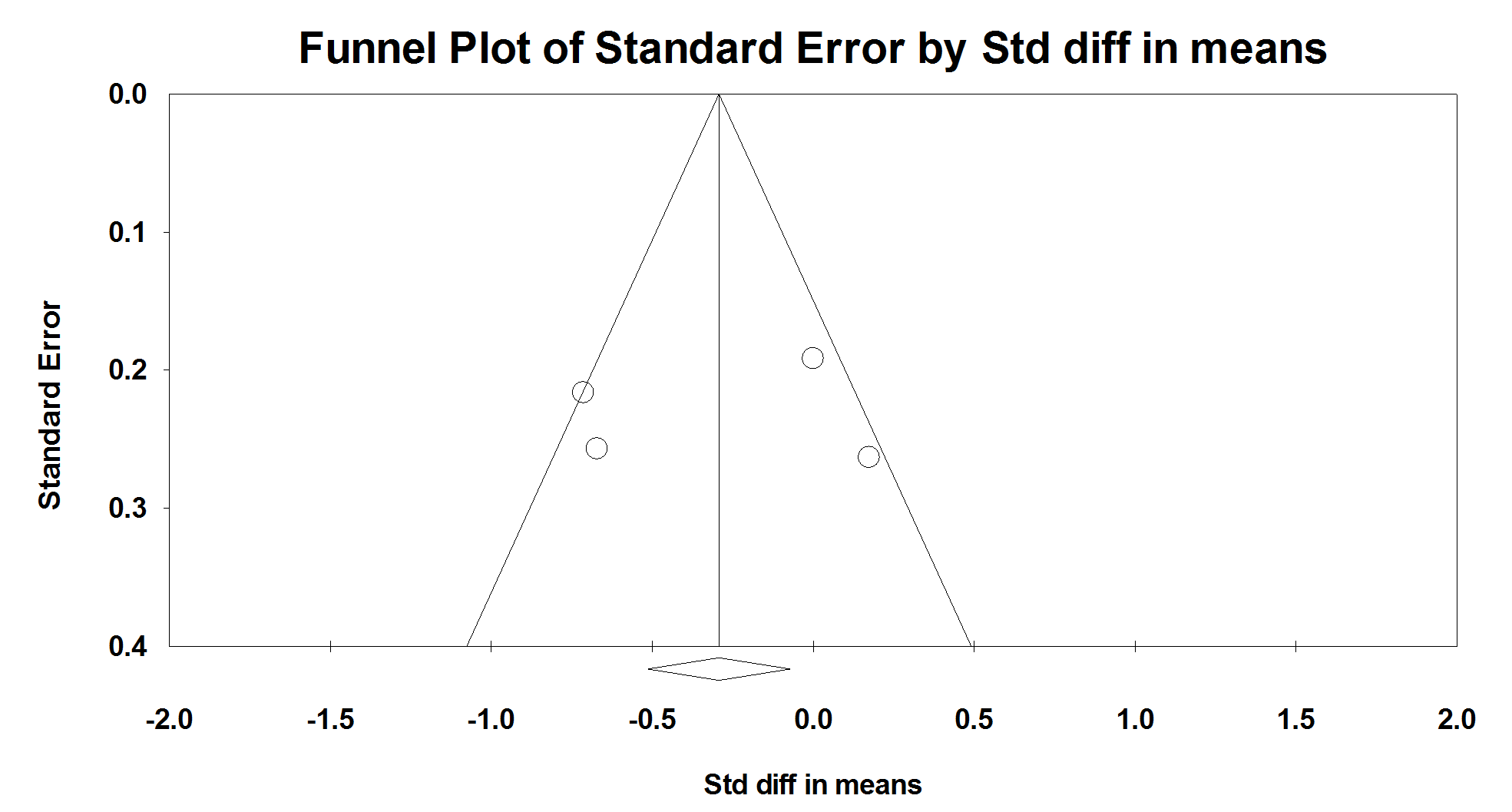

Supplement: Supplementary file 4 — Figure S4. Funnel plot of amount of opioid consumption at 24 h. (TIF 6106 kb) [file 12891_2019_2628_MOESM4_ESM.tif]

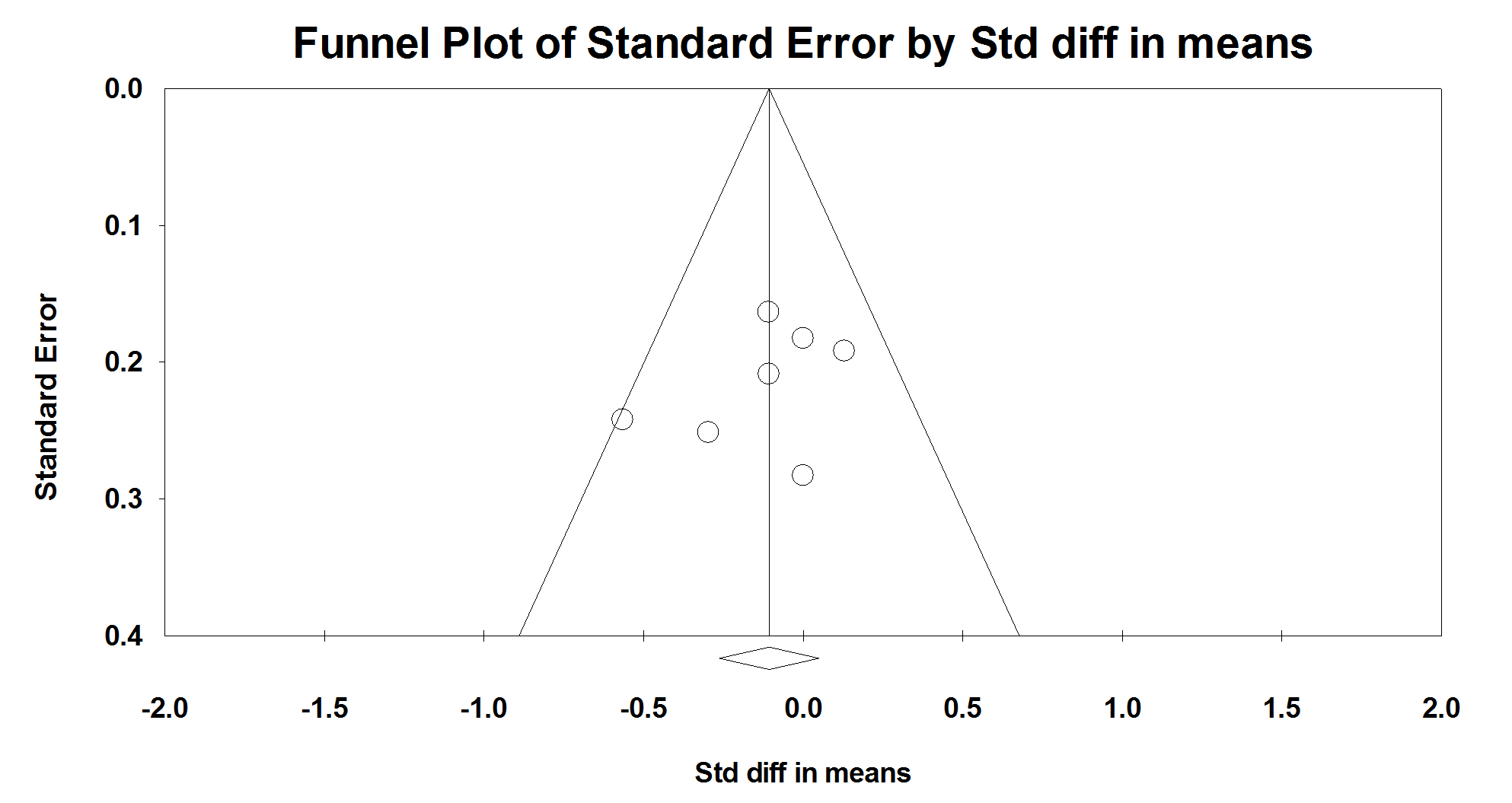

Supplement: Supplementary file 5 — Figure S5. Funnel plot of length of hospital stay. (TIF 6146 kb) [file 12891_2019_2628_MOESM5_ESM.tif]

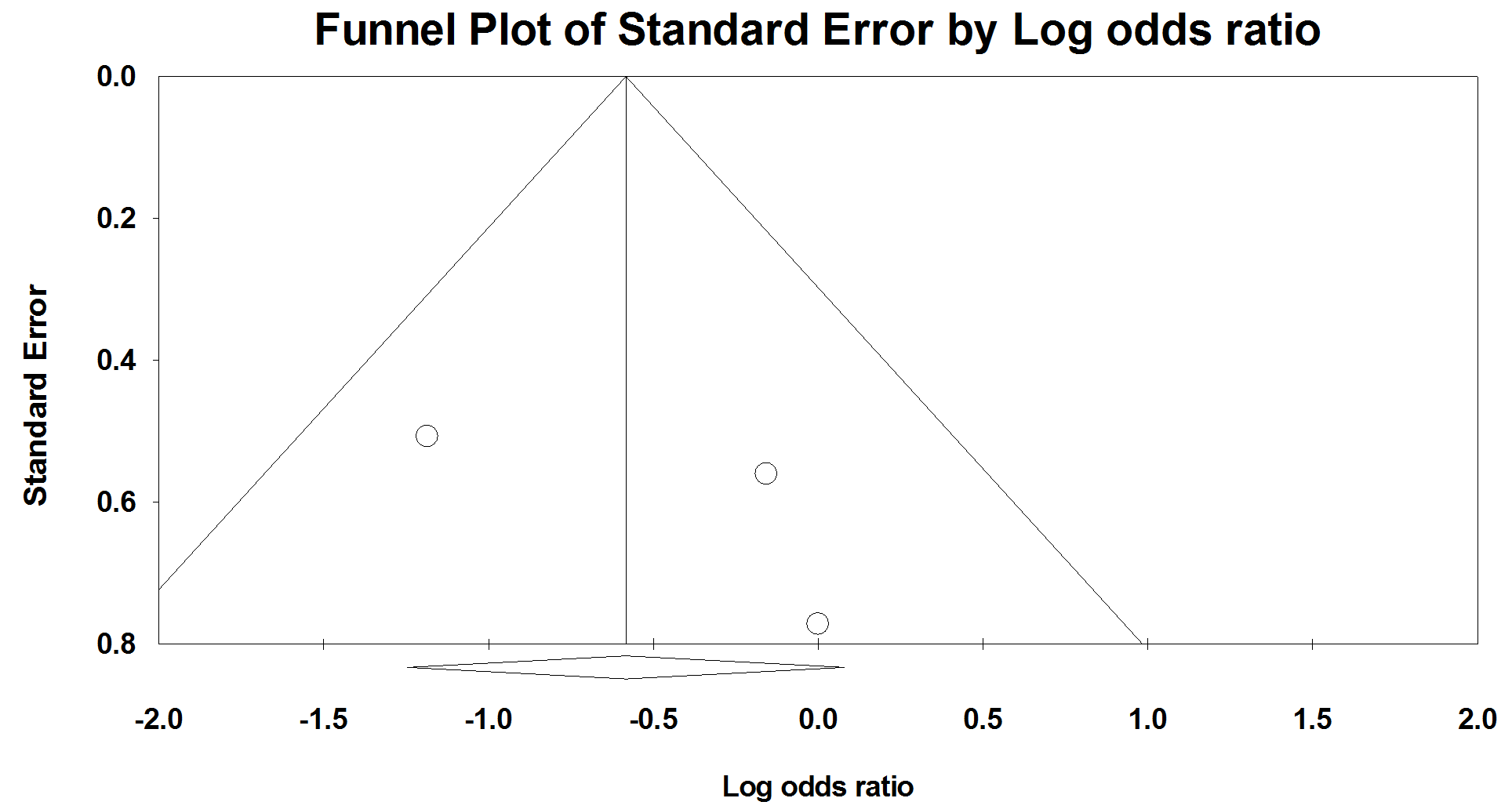

Supplement: Supplementary file 6 — Figure S6. Funnel plot of post-operative nausea. (TIF 5837 kb) [file 12891_2019_2628_MOESM6_ESM.tif]
